# Supplementary material for: Neural correlates of thematic role assignment for passives in Standard Indonesian
Source: PLoS One. 2022 Aug 9;17(8):e0272207. doi: 10.1371/journal.pone.0272207 (PMC9362935; doi:10.1371/journal.pone.0272207)
Supplement: Attachment — Submitted filename: Response to reviewers 2.docx [file pone.0272207.s003.docx]

We would like to express our gratitude to the editor and reviewers for the constructive feedback that has helped improve the manuscript. All changes made are highlighted yellow. Responses to each individual point are provided below. We have also changed the timeline details to match the expected start date of our experiments (p26).

**Reviewer 1**

I appreciate the revisions and think it essentially looks good, but I still am confused about sentence examples (2) and (3) on page 5.

The provided English translations are identical for these two sentences ~"the guitar player discovered the singer", implying that the semantic roles are identical in the two sentences. But, as I understand it, the case marker on guitar player is different (nominative in (2), and accusative in (3)), which implies that the semantic roles should be reversed between (2) and (3), such that the translation for (2) should be as indicated, but the translation for (3) should be something like "the gifted singer discovered the talented guitar player". If so, then this needs to be revised accordingly. Unless I am missing something.

Thank you for pointing this out, we have revised the translation of (3) in p5.

**Reviewer 2**

The authors thoughtfully and satisfyingly addressed the raised concerns and comments. I think that the predictions were more clearly explained and easier to follow in the response than in the manuscript, but I don’t think this necessitates changes to the manuscript.

Overall, I think this will be a very interesting study and wish the best to the authors. I’m looking forward to the future results.

Thank you for the feedback, we have added a part of the discussion from the previous response letter into the predictions to improve the clarity of the subsection (p25).
